# The anabolic response to a ground beef patty and soy-based meat alternative: a randomized controlled trial

**DOI:** 10.1016/j.ajcnut.2024.08.030

**Published:** 2024-08-31

**Authors:** David D Church, Katie R Hirsch, Shiloah A Kviatkovsky, Joseph J Matthews, Arny A Ferrando, Gohar Azhar, Robert R Wolfe

**Affiliations:** 1 Department of Geriatrics, Donald W. Reynolds Institute on Aging, Center for Translational Research in Aging and Longevity, University of Arkansas for Medical Sciences, Little Rock, AR, United States; 2 Department of Exercise Science, Arnold School of Public Health, University of South Carolina, Columbia, SC, United States

**Keywords:** muscle protein synthesis, essential amino acids, protein quality, stable isotope tracers, dietary protein, protein metabolism, plant-based proteins, whole-food protein matrix

## Abstract

**Background:**

Soy-based meat alternatives (SBMA) are becoming increasingly popular, but it is unclear if they have the same anabolic effect on skeletal muscle as animal meat.

**Objectives:**

We aimed to compare the stimulation of skeletal muscle protein synthesis by consumption of 1 or two 4 oz patties of SBMA with 4 oz (80% protein/20% fat) beef.

**Methods:**

The study design was a randomized controlled trial. Participants were aged 18–40 y of age and in good general health with a body mass index (kg/m^2^) between 20 and 32. Stable isotope tracer methods were used (L-[ring-^2^H_5_] phenylalanine, [U-^13^C_9_-^15^N]- tyrosine, and L-[ring-^2^H_4_] tyrosine) to quantify the response of muscle protein fractional synthetic rate (FSR) to consumption of a single beef (4 oz), single SBMA (4 oz), or two 4 oz SBMA patties (8 oz). Whole-body rates of protein synthesis, breakdown, and net balance, as well as plasma essential amino acid concentrations, were also measured.

**Results:**

The increase above basal in muscle protein FSR following consumption of the 4 oz beef patty (0.020 ± 0.016%/h) was significantly greater than the increase following consumption of 4 oz SBMA (*P* = 0.021; 0.003 ± 0.010%/h) but not 8 oz SBMA (*P* = 0.454; 0.013 ± 0.016%/h). The maximal essential amino acid concentration was significantly correlated (*P* = 0.046; r = 0.411) with the change in muscle FSR from the basal to the postprandial period. In addition, the change in muscle FSR from the basal to postprandial period was significantly correlated (*P* = 0.046; r = 0.412) with the corresponding change in whole-body protein synthesis.

**Conclusions:**

Consumption of a 4 oz beef patty stimulates muscle and whole-body protein synthesis >4 oz SBMA patty and similarly to 8 oz of SBMA.

This trial was registered at clinicaltrials.gov as NCT05197140.

## Introduction

Protein ingestion and subsequent hyperaminoacidemia is a robust stimulator of muscle protein synthesis (MPS). MPS, reflected by the rate of muscle protein fractional synthetic rate (FSR), plays a crucial role in the metabolic health of skeletal muscle by renewing older, less functional muscle protein fibers with better-functioning fibers [1]. Essential amino acids (EAAs) are responsible for increasing FSR after consumption of dietary protein, and the appearance of EAAs in the circulation is a primary predictor of anabolism [2]. Increases in plasma EAA are dictated by the content and bioavailability of EAAs in a protein source [3]. Animal-based meat proteins are nutrient-dense whole foods containing all EAAs required for adult human needs [4].

Plant-based meat alternatives (PBMAs) have become increasingly popular among consumers. Recent innovations in formulation and processing operations have led to a new generation of ultra-processed PBMAs designed to mimic the taste, texture, and presentation of meat. Most PBMAs contain equivalent amounts of protein as compared to their meat counterpart. Soy protein is most commonly the primary protein source in PBMA due to its relatively favorable amount and profile of EAAs as compared to other plant-based protein food sources [4]. Nonetheless, the EAA content of soy protein is lower than the same amount of animal meat protein on a gram/gram basis [5]. Further, extensive splanchnic clearance of EAAs absorbed after soy consumption limits the response of peripheral blood concentrations [6,7]. As a result, there may be less stimulation of muscle protein FSR by soy protein consumption as compared to meat protein [8,9], even if whole-body protein net balance (which includes splanchnic uptake) is comparable to that following meat consumption. In contrast to the response to soy protein, beef protein results in a robust stimulation of muscle FSR that is related to a more rapid and greater availability of plasma EAAs than after consumption of soy [10].

In this study, we have investigated the hypothesis that muscle protein FSR is stimulated more after consumption of a 4-oz beef patty (80% protein/20% fat) as compared to 4- and 8-oz of a soy-based meat alternative (SBMA) designed to mimic an 80% protein/20% fat beef patty (Impossible Burger), and that the greater stimulation of muscle FSR after beef consumption is related to differences in the responses of peripheral plasma concentrations of EAAs. We have further investigated the response of whole-body protein kinetics to the consumption of the 2 types of burgers to test the hypothesis that EAA uptake for muscle protein FSR after beef consumption represents a greater proportion of total protein intake than after consumption of a soy-based PBMA.

## Methods

### Participants

Twenty-four participants were aged 18–40 y of age (mean ± SD: 31.8 ± 6.7 y) and in good general health with a BMI between 20 and 32 (Table 1). The intervention experimental test days were conducted between 3 June, 2022 and 3 November, 2022. Screening included a standard battery of medical tests, including medical history, blood count, and medical questionnaires. Participants were excluded from participation if they: *1*) had complete blood count laboratory results that indicated anemia or abnormal white blood cell counts; *2*) had a history of chemotherapy or radiation in the 6 mo prior to enrollment; *3*) were using insulin to control blood glucose concentrations; *4*) were currently receiving androgen (e.g., testosterone) or anabolic (e.g., growth hormone, insulin-like growth factor 1) therapy; *5*) were currently using prescription blood thinning medications; or *6*) were unable or unwilling to consume animal protein sources (i.e., vegan or vegetarian). Written informed consent was obtained from all participants, and the study was approved by the institutional review board at the University of Arkansas for Medical Sciences and was registered at clinicaltrials.gov (NCT05197140). Subjects were compensated to cover travel expenses. No important changes in health status occurred after the trial started.

**TABLE 1:** Participant demographics.

Characteristic	4 oz beef	4 oz SBMA	8 oz SBMA
Age (y)	30.0 ± 7.4	36.5 ± 4.2	28.9 ± 5.8
Males/females (*n*)	4/4	3/5	3/5
Body mass (kg)	81.2 ± 14.0	79.1 ± 12.6	78.8 ± 11.9
BMI (kg/m^2^)	27.6 ± 3.9	27.0 ± 3.6	27.3 ± 2.9
Fat mass (kg)	27.0 ± 6.7	26.5 ± 6.4	25.1 ± 6.8
Fat-free mass (kg)	53.6 ± 8.6	50.7 ± 13.0	52.0 ± 9.6
Skeletal muscle mass (kg)	31.4 ± 5.4	29.1 ± 8.4	30.3 ± 6.2

Abbreviations: BMI, body mass index; SBMA, soy-based meat alternative; SD, standard deviation.

Values are presented as mean ± SD. *N* = 8 subjects per group.

### Experimental design

Participants were randomly assigned to 1 of 3 intervention groups via a single-blinded (outcome assessor and principal investigator) randomization, stratified for sex: 4 oz beef patty (80% protein, 20% fat); 4 oz SBMA patty; 2 × 4 oz (8 oz) SBMA patties (*n* = 8 participants per group). The randomization procedure to allocate the treatment group was executed via a random-number generator (www.randomization.com). The Impossible Burger was selected as a representative PBMA as soy is the primary source of protein, which is a high-quality plant protein, and the Impossible Burger was specifically designed to mimic the tastes and texture of a beef burger. The nutritional information of the 3 options is shown in Table 2 [11]. The burgers were precooked and frozen in marked packages, and the appropriate burger was thawed and heated in a microwave prior to each individual study.

**TABLE 2:** Macronutrient content of 3 meal options.

Nutrient	4 oz Beef	4 oz SBMA	8 oz SBMA
Calories (kcal)	279	231	462
Protein (g)	27.3	20.5	40.9
∑EAA (g)	10.2	7.5	15
Leucine (g)	1.96	1.53	3.06
Carbohydrates (g)	0	9	18
Sugars (g)	0	<1	<1
Fats (g)	18	13	26
Saturated fats (g)	8	6	12

Abbreviations: EAA, essential amino acids; SBMA, soy-based meat alternative; USDA, United States Department of Agriculture.

Protein and amino acid values obtained from reference Fanelli et al. [11]. The remaining values were obtained from USDA (usda.gov). The full ingredients list is provided in Supplemental Table 1.

Body composition was determined from a dual-energy x-ray absorptiometry whole-body scan (GE Lunar iDXA; GE Medical Systems Ultrasound & Primary Care Diagnostics) and bioelectrical impedance analysis (InBody770; BioSpace). Although this approach provides an imprecise estimation of skeletal muscle mass, conclusions were not affected because there were no changes during the trial or differences between groups.

#### Stable isotope tracer infusion protocol

Subjects reported to the research clinic at 07:00 after an overnight fast from 22:00 the previous night and participated in a 2-period tracer infusion-metabolic study utilizing stable isotope tracers: 4 h for the baseline fasted period and 6 h for the postmeal period (total 10-h period). MPS was measured directly by tracer incorporation [12], and whole-body protein kinetics were determined by stable isotope tracer methodology using the “bioavailability” method, as discussed in detail in reference [13].

All data collection occurred at the University of Arkansas for Medical Sciences Center for Translational Research in Aging and Longevity. Upon arrival, catheters were inserted into a vein in the forearm of 1 arm for tracer infusion and in a hand or wrist vein of the contralateral arm for blood sampling using the heated hand technique. After obtaining a blood sample to determine background enrichments, priming doses of L-[ring-^2^H_5_] phenylalanine (Phe; 3.6 μmol/kg), [U-^13^C_9_-^15^N]- tyrosine (Tyr; 0.113 μmol/kg), and L-[ring-^2^H_4_] Tyr (0.3 μmol/kg) was given. Infusions of L-[ring-^2^H_5_] Phe (3.6 μmol/kg/min) and U-^13^C_9_-^15^N- Tyr (0.113 μmol/kg/min) were then started and maintained throughout the metabolic study. Baseline blood samples were taken before the start of the tracer infusion. Postabsorptive samples were obtained at 120, 150, 180, 210, and 240 min. After the final fasted blood sample was drawn, subjects consumed 4 oz beef patty, 4 oz SBMA, or 8 oz SBMA, depending on the group to which they were randomly assigned. Blood samples were drawn at 270, 300, 330, 360, 390, 420, 450, 480, 510, 540, 570, and 600 min of tracer infusion (postingestion blood samples) to measure tracer enrichment (Supplemental Figure 1) and plasma responses of EAAs and insulin. Muscle biopsies were obtained before the start of the infusion, at 240 min to obtain baseline muscle FSR and at 600 min to determine postprandial muscle protein FSR.

#### Calculation of protein kinetics

Muscle protein FSR was calculated by dividing the increase in tracer enrichment in skeletal muscle protein over time by the precursor enrichment, taken to be the intracellular free Phe enrichment [12]. In order to estimate the percentage of MPS responsible for whole-body protein synthesis (described below), the postprandial period FSR was multiplied by the measured skeletal muscle mass, of which 20% was assumed to be protein [14] and expressed as the total response over the 6-h postprandial period.

The calculation of whole-body protein kinetics (protein synthesis, protein breakdown, and net protein balance) is based on the determination of the rate of appearance (Ra) of Phe and of Tyr into plasma and the fractional Ra of endogenous Tyr resulting from Phe hydroxylation. We have previously described and discussed in detail the 2-pool model we used [13]. Briefly, an isotopic steady state was established in the baseline/fasted period, and protein kinetics were calculated accordingly [12]. The AUC of plasma enrichments of Phe and Tyr tracers were calculated for 6 h after ingestion of the patty. Ra of Phe reflects protein breakdown in the fasted state; the total appearance of Phe over the 6 h postprandial state reflects both protein breakdown and the appearance of protein from the ingested meal. The appearance of exogenous Phe in the peripheral circulation must be subtracted from the total appearance of Phe to determine the rate of endogenous protein breakdown. The total appearance of exogenous Phe in peripheral blood is estimated from the amount of Phe in the dietary protein and the amount of protein consumed, the published value for the true ileal digestibility of Phe in the test protein [11], and the measured fraction of absorbed Phe hydroxylated to Tyr. We have recently discussed in detail the assumptions underlying this model of protein kinetics [13].

#### Analytic methods

Plasma samples were processed as previously described for determination of enrichment by GC-MS [12]. Plasma amino acid concentrations were determined by liquid chromatography-mass spectrometry using the internal standard method as described previously [15].

### Statistical analysis

We performed a sample size calculation for a 1-way analysis of variance (ANOVA) F-test to compare plasma EAA AUC between 3 treatment groups based on previous studies comparing equal servings of soy and beef [5]. Using G∗Power software [16] (version 3.1.9.7), we found that with a significance level (α) of 0.05 and an effect size (f) of 0.80, a sample size of *n* = 9 per treatment group would be sufficient to detect a difference in plasma EAA AUC of EAA concentration compared with time with a power of >0.8. Further, analysis of existing muscle FSR data comparing similar treatment groups [16] indicated similar size groups were required to observe a significant difference in muscle FSR.

The primary outcome was the plasma EAA concentration AUC above baseline (AUC_i_) over the 6 h after consumption of the protein food source; muscle protein FSR and whole-body protein kinetics were secondary outcomes 1-way ANOVA was used to compare differences in protein kinetics (muscle protein FSR, whole-body net balance, protein synthesis, and protein breakdown). In addition, 2 [state (postabsorptive compared with postprandial)] by 3 [group (4 oz beef compared with 4 oz SBMA compared with 8 oz SBMA)] ANOVA was used to compare the temporal change in muscle FSR and whole-body protein metabolism across groups. One-way ANOVAs were analyzed on the AUC_i_, postingestion maximal concentration (C_max_), change from baseline to C_max_ [delta(Δ)], the time to C_max_, and rate to C_max_ for the sum of the EAAs, sum of the branched-chain amino acids (BCAAs), leucine, and Phe. Values are presented as mean ± SD. No changes to trial outcomes or interim analysis occurred after the trial commenced. All significant main effects of the group were followed by Tukey pairwise comparisons. Normal distribution of the data was confirmed by the Shapiro-Wilk test and visual inspection of Q-Q plots. In some analyses, 1 group of data was not normally distributed (4 oz SBMA plasma EAA AUC_i_ and EAA concentration), but the ANOVA error rate is considered robust to such deviations [17]. Pearson correlation (r) was used to assess the relationship between changes in muscle FSR and changes in EAA concentration and whole-body protein synthesis. Statistical significance was accepted at *P* < 0.05.

## Results

### Participant characteristics

**FIGURE 1:**
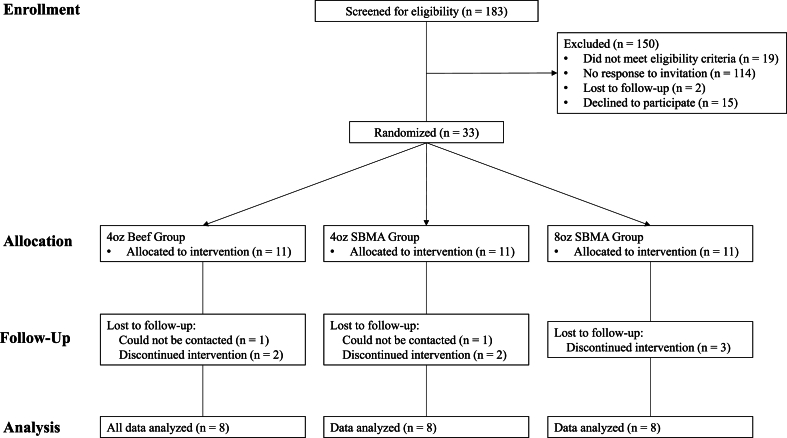
CONSORT flow diagram. CONSORT, consolidated standards of reporting trials; SBMA, soy-based meat alternative.

The baseline characteristics of the participants who were randomly assigned to the intervention arms and completed the trial are presented in Table 1. A CONSORT flow diagram is shown in Figure 1. Due to noncompleters, each group contains data for *n* = 8 participants.

### Muscle protein FSR

The repeated 2 by 3 measures ANOVA indicated a significant phase-by-treatment interaction (*P* = 0.020). Post hoc testing indicated muscle protein FSR was significantly elevated above the basal value for the 4 oz beef burger (basal 0.035 ± 0.008%/h, postprandial 0.055 ± 0.010%/h; *P* = 0.008) and 8 oz SBMA (basal 0.037 ± 0.008%/h, postprandial 0.050 ± 0.010%/h; *P* = 0.003). In contrast, 4 oz SBMA did not stimulate muscle protein FSR above the basal value (basal 0.041 ± 0.009%/h, postprandial 0.044 ± 0.011%/h; *P* = 0.435).

**FIGURE 2:**
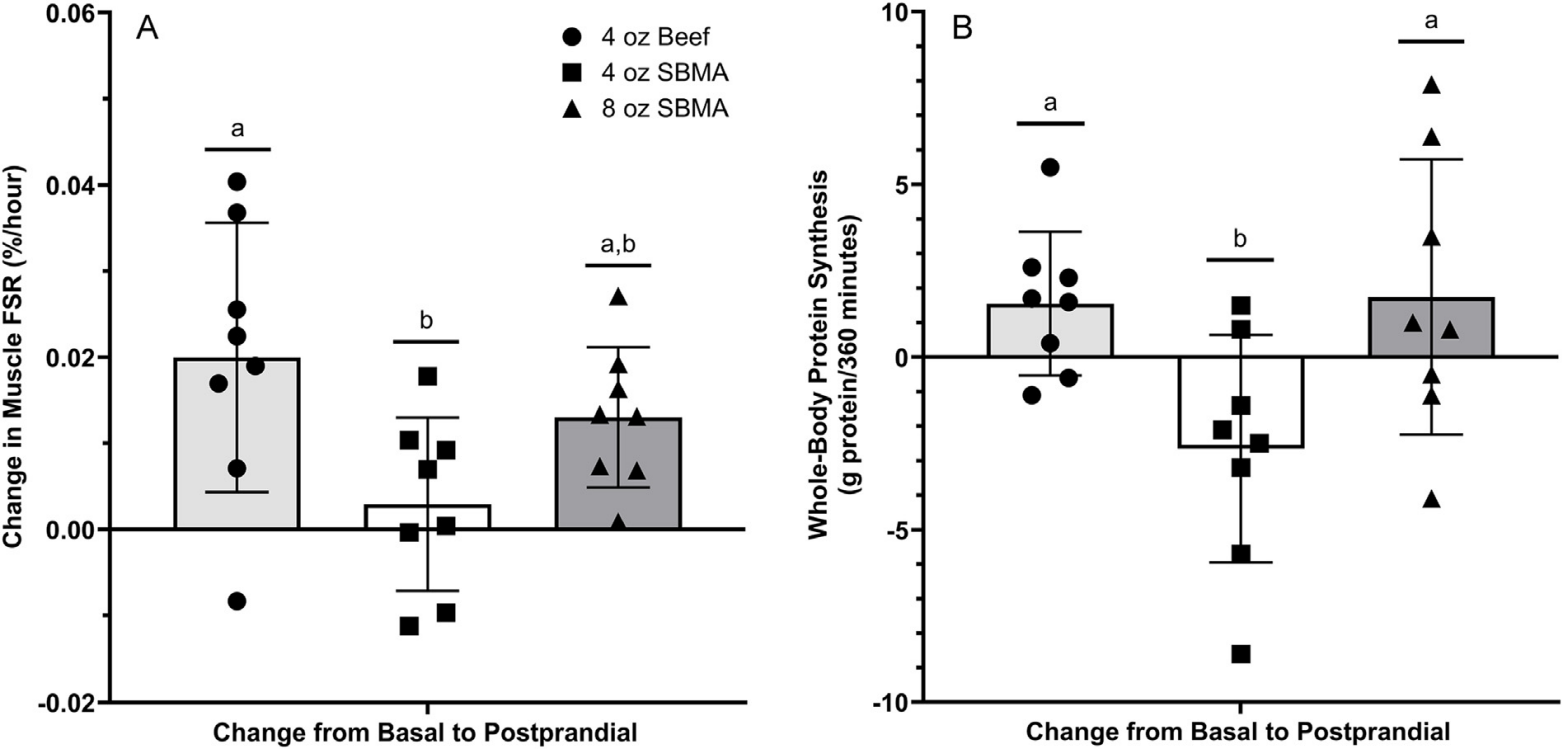
Muscle fractional synthesis rate (A) FSR and whole-body protein synthesis (B). Groups not sharing a similar letter are significantly different from 1 another. FSR, fractional synthetic rate; SBMA, soy-based meat alternative.

The 1-way ANOVA on the increase in muscle protein FSR from basal to postabsorptive indicated a significant main effect of group (*P* = 0.026), with the beef group (0.020 ± 0.016%/h) being significantly >4 oz SBMA group (0.003 ± 0.010%/h; *P* = 0.021) but not the 8 oz SBMA group (0.013 ± 0.016%/h; *P* = 0.454). Further, the 8 oz SBMA group was not significantly different (*P* = 0.223) than the 4 oz SBMA group (Figure 2).

A 1-way ANOVA indicated a trend toward a group difference (*P* = 0.052) for the contribution of MPS to whole-body protein synthesis. Post hoc test indicated that one 4 oz beef patty resulted in a significantly (*P* = 0.041) greater contribution of MPS to whole-body protein synthesis (35% ± 6%) as compared to 4 oz SBMA (26% ± 8%), but not 8 oz SBMA (30% ± 7%; *P* = 0.339). No significant differences (*P* = 0.482) were noted between the 2 SBMA groups.

### Whole-body protein synthesis

The repeated 2 by 3 measures ANOVA indicated a significant phase-by-treatment interaction (*P* = 0.020). Post hoc testing indicated a trend for the whole-body rate of protein synthesis in the postprandial state to be elevated as compared to the basal period (*P* = 0.074) in the beef burger group. Further, a trend for a decrease (*P* = 0.056) was observed for the 4 oz SBMA group, whereas the 8 oz SBMA group showed no significant difference between the basal and postprandial state (*P* = 0.262). A 1-way ANOVA on the Δ scores indicated a significant between-group difference (*P* = 0.020). Post hoc test indicated the change in whole-body protein synthesis from basal to postprandial was significantly greater in the beef (1.6 ± 2.1 g protein/360 min; *P* = 0.042) and the 8 oz SBMA group (1.7 ± 4.0 g protein/360 min; *P* = 0.033) as compared to the 4 oz SBMA (–2.6 ± 3.3 g protein/360 min) (Figure 2).

### Whole-body protein breakdown

The 1-way ANOVA on the change in protein breakdown from the basal to postprandial indicated significant (*P* ≤ 0.001) between-group effects. Post hoc testing indicated all groups to be significantly different in the change in protein breakdown from the basal to postprandial state (*P* ≤ 0.001–0.043). The repeated 2 by 3 measures ANOVA indicated a significant phase-by-treatment interaction (*P* ≤ 0.001). Post hoc testing indicated that all groups significantly (*P* ≤ 0.001) attenuated protein breakdown in the postprandial state. Significant group differences were noted in the postprandial period (*P* = 0.002) but not in the basal state (*P* = 0.980). Post hoc testing indicated protein breakdown in the postprandial state was significantly lower in the 8 oz SBMA group (20.1 ± 12.9 g protein/360 min) as compared to the beef group (42.1 ± 11.1 g protein/360 min; *P* = 0.003) and the 4 oz SBMA group (39.4 ± 10.5 g protein/360 min; *P* = 0.008), with no difference between the beef and 4 oz SBMA groups.

### Whole-body protein balance

A 1-way ANOVA indicated group differences in the postprandial period (*P* ≤ 0.001) but not in the basal state (*P* = 0.860). Post hoc testing indicated protein balance was significantly greater in the 8 oz SBMA (40.3 ± 4.0 g protein/360 min) as compared to the beef (16.3 ± 2.6 g protein/360 min; *P* ≤ 0.001) and 4 oz SBMA groups (17.3 ± 2.5 g protein/360 min; *P* ≤ 0.001) in the postprandial state. No difference was noted for protein balance in the postprandial state between the beef and 4 oz SBMA groups (*P* = 0.783). The repeated 2 by 3 measures ANOVA indicated a significant phase-by-treatment interaction (*P* ≤ 0.001). Post hoc testing indicated that all groups significantly (*P* ≤ 0.001) improved protein balance in the postprandial state.

### Phe hydroxylation

One-way ANOVA’s indicated no significant group differences in the basal (*P* = 0.879; 0.05 ± 0.01 *μ*mol/kg/min) and postprandial (*P* = 0.427; 0.06 ± 0.01 *μ*mol/kg/min) states. Further, a 1-way ANOVA on hydroxylation indicated no significant group differences (*P* = 0.569; 0.07 ± 0.01 *μ*mol/kg/min). The repeated 2 by 3 measures ANOVA indicated a significant phase-by-treatment interaction (*P* = 0.024). Post hoc testing indicated that all groups significantly (*P* ≤ 0.001–0.020) increased hydroxylation in the postprandial state.

### Plasma amino acid pharmacokinetics

**FIGURE 3:**
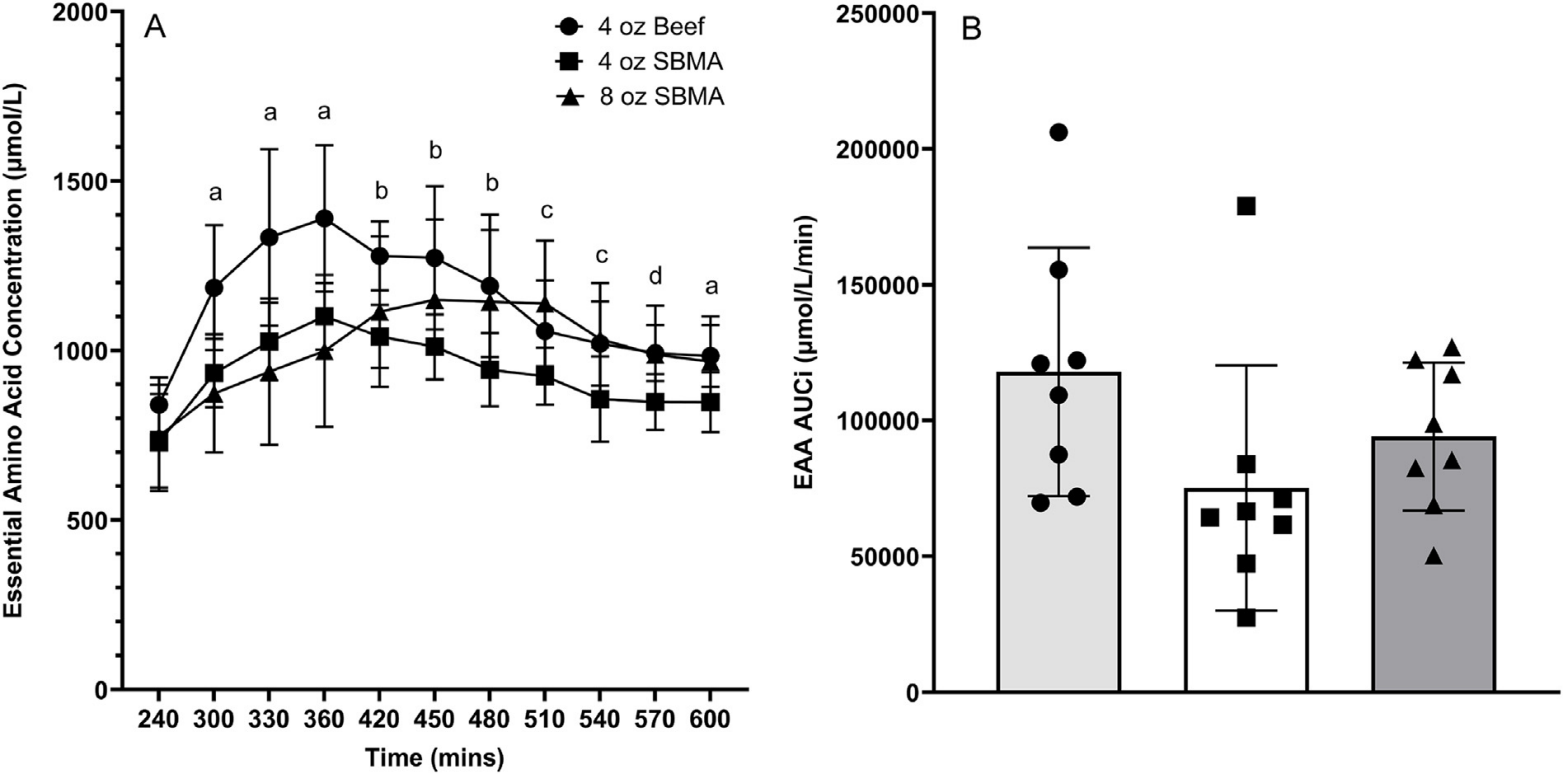
Plasma essential amino acid concentration responses vs time (A) and area under the curve above baseline (B [AUCi]). a4 oz beef significantly greater than 4 and 8 oz SBMA. b4 oz beef significantly greater than 4 oz SBMA. c8 oz SBMA significantly greater than 4 oz SBMA. d4 oz beef and 8oz SBMA significantly greater than 4 oz SBMA. SBMA = soy-based meat alternative.

The EAA response over time and the AUC of the total postprandial EAA responses are shown in Figure 3. For the EAAs, the 1-way ANOVA indicated significant between-group differences for C_max_ (*P* ≤ 0.001), Δ EAA (*P* ≤ 0.001), time to C_max_ (*P* = 0.002), and rate to C_max_ (*P* = 0.025), but not AUC_i_ (*P* = 0.129). For the BCAAs, the 1-way ANOVA indicated significant between-group differences for C_max_ (*P* ≤ 0.001), Δ BCAA (*P* = 0.023), time to C_max_ (*P* = 0.005), and rate to C_max_ (*P* = 0.019), but not AUC_i_ (*P* = 0.153). For leucine, the 1-way ANOVA indicated significant between-group differences for C_max_ (*P* ≤ 0.001), Δ (*P* = 0.004), time to C_max_ (*P* = 0.009), and rate to C_max_ (*P* = 0.012), and a trend for AUC_i_ (*P* = 0.055). For Phe, the 1-way ANOVA indicated significant between-group differences for time to C_max_ (*P* = 0.004), but not AUC_i_ (*P* = 0.162), C_max_ (*P* = 0.232), Δ (*P* = 0.257), rate to C_max_ (*P* = 0.475). Post hoc testing results are presented in Table 3.

**TABLE 3:** Plasma amino acid pharmacokinetics.

Amino acid	Condition	AUC_i_ (μmol/L/min)	Max concentration (μmol/L)	Δ concentration (μmol/L)	Time to maximal concentration (min)	Rate to maximal concentration (μmol/L/min)
∑EAA	4 oz Beef	117,896 ± 45,786	1529 ± 116^a^	689 ± 164^a^	142 ± 59^a^	5.6 ± 2.5^a^
	4 oz SBMA	75,097 ± 45,231	1126 ± 89^b^	397 ± 100^b^	131 ± 45^a^	3.8 ± 2.8^a^
	8 oz SBMA	94,057 ± 27,252	1216 ± 197^b^	469 ± 131^b^	228 ± 50^b^	2.2 ± 1.0^b^
∑BCAA	4 oz Beef	66,878 ± 23,201	718 ± 58^a^	348 ± 90^a^	150 ± 60^a^	2.6 ± 1.1^a^
	4 oz SBMA	48,315.6 ± 16,091	550 ± 39^b^	237 ± 41^b^	146 ± 37^a^	1.7 ± 0.5^a,b^
	8 oz SBMA	64,336 ± 19,619	643 ± 111^b^	317 ± 87^a,b^	240 ± 68^b^	1.5 ± 0.6^b^
Leucine	4 oz Beef	24,299 ± 8351	241 ± 20^a^	130 ± 30^a^	150 ± 60^a^	1.0 ± 0.4^a^
	4 oz SBMA	15,857 ± 4685	174 ± 13^b^	80 ± 13^b^	146 ± 37^a^	0.6 ± 0.2^b^
	8 oz SBMA	21,411 ± 6382	208 ± 40^a,b^	109 ± 30^a,b^	232 ± 67^b^	0.5 ± 0.2^b^
Phenylalanine	4 oz Beef	3,571.8 ± 2392	84 ± 11	25 ± 7	142 ± 67^a^	0.2 ± 0.1
	4 oz SBMA	4,319.9 ± 2564	76 ± 8	24 ± 5	131 ± 31^a^	0.2 ± 0.1
	8 oz SBMA	5932 ± 2297	90 ± 23	30 ± 10	221 ± 47^b^	0.2 ± 0.1

Abbreviations: AUC_i_, area under the curve above baseline; BCAA, branched-chain amino acid; EAA, essential amino acid; SBMA, soy-based meat alternative; SD, standard deviation.

Values are presented as mean ± SD.

Values not sharing the same letter are significantly different from 1 another (*P* < 0.05).

### Correlations

**FIGURE 4:**
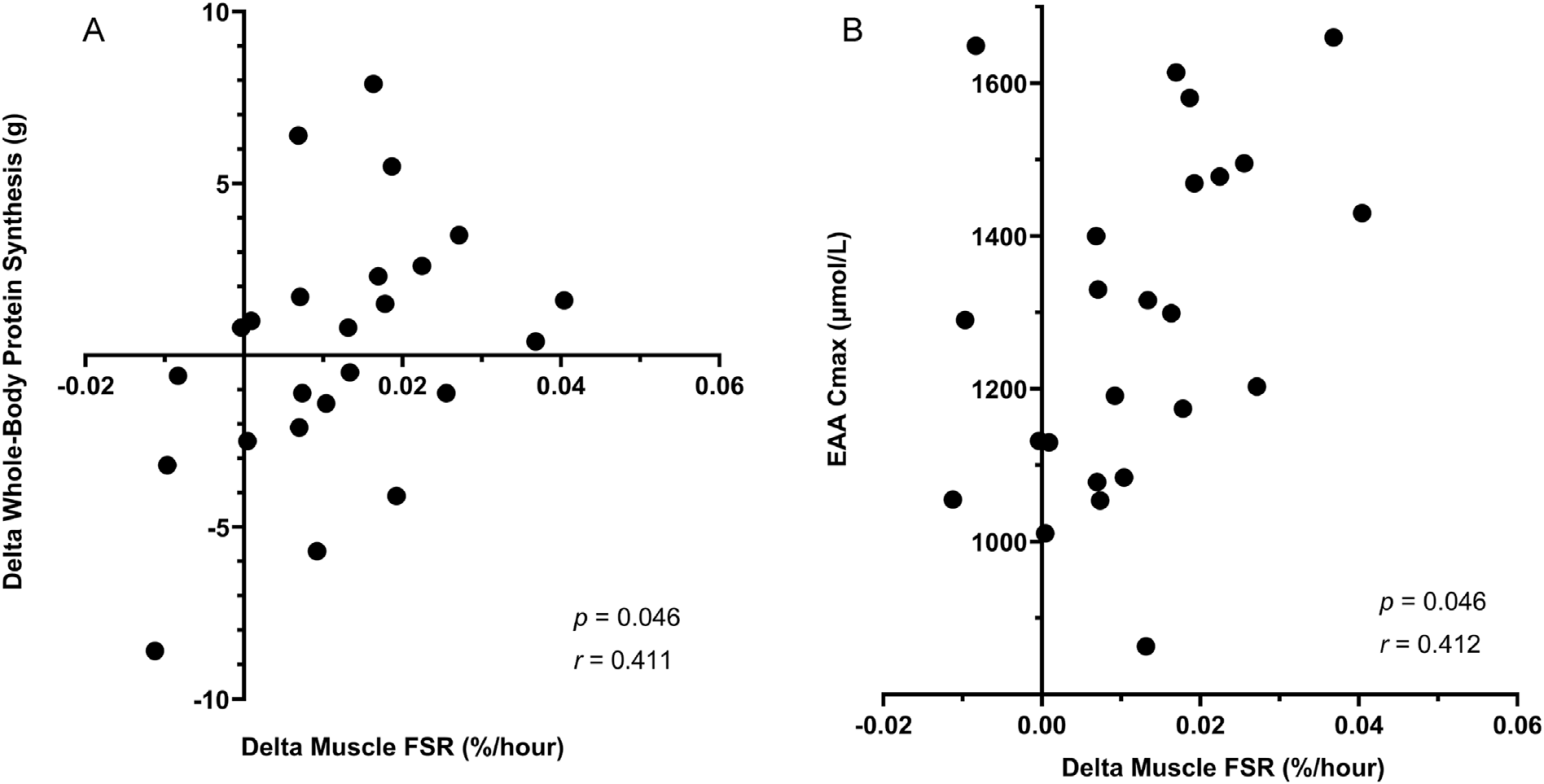
Correlations between change in muscle protein fractional synthetic rate (FSR) and whole-body protein synthesis (A) and change in muscle FSR and maximal plasma essential amino acid (EAA) concentration (B).

In accordance with our previous results [2], the maximal EAA concentration (C_max_) was significantly correlated (*P* = 0.046; r = 0.411) with the change in muscle FSR from the basal to the postprandial period (Figure 4). In addition, the change in muscle FSR from the basal to postprandial period was significantly correlated (*P* = 0.046; r = 0.412) with the change in whole-body protein synthesis from the basal to postprandial period Figure 4.

## Discussion

The principal finding of this study is that consumption of a beef patty stimulated MPS significantly more than consumption of an SBMA patty (Figure 2). Stimulation of muscle protein FSR is physiologically important for the renewal of muscle fibers, irrespective of any changes in total muscle protein mass [1]. The greater stimulation of MPS resulting from consumption of a beef patty, as measured by direct incorporation of amino acid tracer into muscle protein, was consistent with the increase in whole-body protein synthesis and represented a greater proportion of ingested dietary protein than when a single SBMA patty was consumed. The muscle protein responses were related to corresponding increases in plasma EAA concentrations (FIGURE 2, FIGURE 4).

The physiologic basis for the greater anabolic effect of beef on muscle protein is not certain. Much attention has been given to the “leucine trigger” concept, whereby changes in leucine concentration are key in activating MPS [18]. The beef patty contained more leucine, as well as total EAAs, than the SBMA. However, the proportion of leucine in the EAAs in the food, as well as the plasma EAA concentration response, was similar between beef and SMBA. In fact, the 2 SBMA patties had 50% more leucine than the single beef patty; however, the stimulation of protein synthesis by 2 SMBA patties was similar to the response to 1 beef patty. It, therefore, seems unlikely that the amount of leucine, as opposed to the total EAAs, was responsible for the greater anabolic response to beef. This perspective is consistent with a recent publication that challenges the “leucine trigger” concept [19].

Differences in splanchnic clearance of absorbed EAAs may have been important in contributing to different responses of muscle protein FSR. Previously modeled responses to soy compared with milk protein estimated a 30% greater splanchnic utilization of the amino acids from soy protein, which resulted in a 20% smaller increase in peripheral protein synthesis [6,7]. The differences in splanchnic and peripheral responses to milk compared with soy protein may reflect the responses to beef compared with soy protein as well. Differences in both composition and splanchnic clearance of beef compared with soy protein would favor the more rapid increase in peripheral EAA concentrations after beef ingestion, as well as a greater total increase (AUC) in plasma EAAs (Figure 3). The more rapid increase and higher peak concentrations of EAAs following beef ingestion provided a greater stimulus for MPS [2,20].

It was striking that consumption of a single SBMA patty failed to stimulate muscle protein FSR even though soy protein is generally considered to be the highest quality plant-based protein [21]. Previous reported observations have indicated that 4 oz of beef elicits a greater MPS response as compared to 4 oz of soy in middle-aged males at rest and postresistance exercise [22]. The lack of stimulatory effect of soy protein on MPS has been previously reported. Yang et al. [8] found that ingestion of either 20 or 40 g of soy protein isolate failed to increase MPS in elderly subjects at rest. Because even a very large dose of soy protein isolate alone failed to provide a significant stimulus for MPS [8], it is possible that the stimulation of MPS by consumption of 2 SBMA patties that we observed was due to the concomitant energy intake. Both carbohydrates [23] and fat [24] may amplify the response of MPS to protein or amino acid consumption. Although the MPS response to 8 oz SBMA was similar to the response to 4 oz beef patty, the consumption of 8 oz SBMA has the disadvantage of containing ∼60% more calories when compared to the beef patty (Table 2).

The measurement of the response of whole-body protein kinetics to dietary protein consumption is not without controversy [13,25,26]. The controversial issue is how to account for the contribution of the exogenous protein to the endogenous amino acid flux representative of protein breakdown. Our approach assumes the contribution of exogenous protein is equal to the amount of protein ingested, corrected for true ileal digestibility. Because the digestibility of beef and soy protein is high and similar [11], it is unlikely our assumption had a significant impact on the comparative results between the 2 protein food sources. Most importantly, the exogenous contribution to amino acid flux is only relevant for the calculation of protein breakdown and net balance. The rate of whole-body protein synthesis does not distinguish the origin of the amino acid tracee and is calculated the same for all tracer infusion methods [13]. Thus, our observation that whole-body protein synthesis increased after beef consumption compared to soy is independent of the tracer method employed.

There are some potential limitations to the present study. First, the macronutrient contents were not the same between treatments. Exactly matching protein, carbohydrate, and fat contents in dietary food sources is impractical with whole-food formats. The use of a whole-food protein format is novel as the majority of protein metabolism research involves protein isolate or other drink formats [e.g., 2,3,8,9,27]. We compared the beef treatment to an amount of SBMA that had lower and higher amounts of calories, protein, EAAs, and leucine. In line with our results, it has been reported that MPS was 47% higher following ingestion of an omnivorous mixed meal as compared to a vegan meal [28]. Thus, we are confident that the lack of an exact macronutrient match does not skew our results. Second, only younger individuals were studied. However, the difference between the response of MPS to lower compared with higher plasma concentrations of EAAs is more pronounced in older as compared to younger individuals [29,30], so the differences in the response to beef compared with SBMA may have been even larger in older subjects than what we are reporting for younger individuals. Lastly, to evaluate the effects of the treatments on selected outcomes, the measurements were performed in the overnight postabsorptive state. Therefore, whether the effects reported would occur in the fed state is unclear.

In conclusion, ingestion of 4 oz of beef stimulates muscle protein FSR >4 oz of SBMA. There were no differences in the stimulation of muscle protein FSR between 4 oz of beef and 8 oz of SBMA. Similar results were noted for whole-body protein synthesis and plasma EAA responses, although 8 oz of SBMA stimulated whole-body protein balance >4 oz of beef or SBMA. Further, the change in the muscle protein FSR response was significantly correlated with the maximal EAA concentration measured following consumption. SBMA can stimulate protein synthesis when enough is consumed, but the corresponding caloric content exceeds that contained in a 4 oz serving of (80/20) beef.

## Author contributions

The authors’ responsibilities were as follows – DDC, KRH, AAF, RRW: conception of the idea, planning of the experiments, and interpretation of results; DDC, KRH, SAK, GA: assisted with data collection; DDC: led the data analysis; DDC, KRH: original drafting of the manuscript; KRH, SAK, JJM, GA, AAF, RRW: reviewed, edited, and provided critical feedback on the final manuscript; and all authors: read and approved the final manuscript.

## Conflict of interest

DDC has performed free-lance work for Soy Connection, funded by the United States Soy and the United Soybean Board, and received research grants from the National Cattlemen’s Beef Association. AAF and RRW have received research grants from the National Cattlemen’s Beef Association. RRW is an owner of The Amino Company and holds several patents of essential amino acid-based nutritional supplements. All other authors report no conflicts of interest.

## Funding

The project was financially supported by a grant from the National Cattleman’s Beef Association. The study sponsor has no role in the design, execution, interpretation, or writing of the study. DDC is currently supported by an NIH Clinical Research Loan Repayment Award. DDC salary was partially supported by the National Center for Advancing Translational Sciences of the NIH under award numbers (TL1 TR003109) and (UL1 TR003107) during the conduct of the trial. The content is solely the responsibility of the authors and does not necessarily represent the official views of the NIH. There are no relationships or activities that could appear to have influenced the submitted work. The findings are presented clearly, honestly, and without fabrication, falsification, or inappropriate data manipulation.

## Data availability

Data described in the manuscript will be made available upon request pending application to and approval from the corresponding author.
