# Supplementary material for: The anabolic response to a ground beef patty and soy-based meat alternative: a randomized controlled trial
Source: Am J Clin Nutr. 2024 Aug 31;120(5):1085–92. doi: 10.1016/j.ajcnut.2024.08.030 (PMC11600063; doi:10.1016/j.ajcnut.2024.08.030)
Supplement: multimedia component 1 [file mmc1.docx]

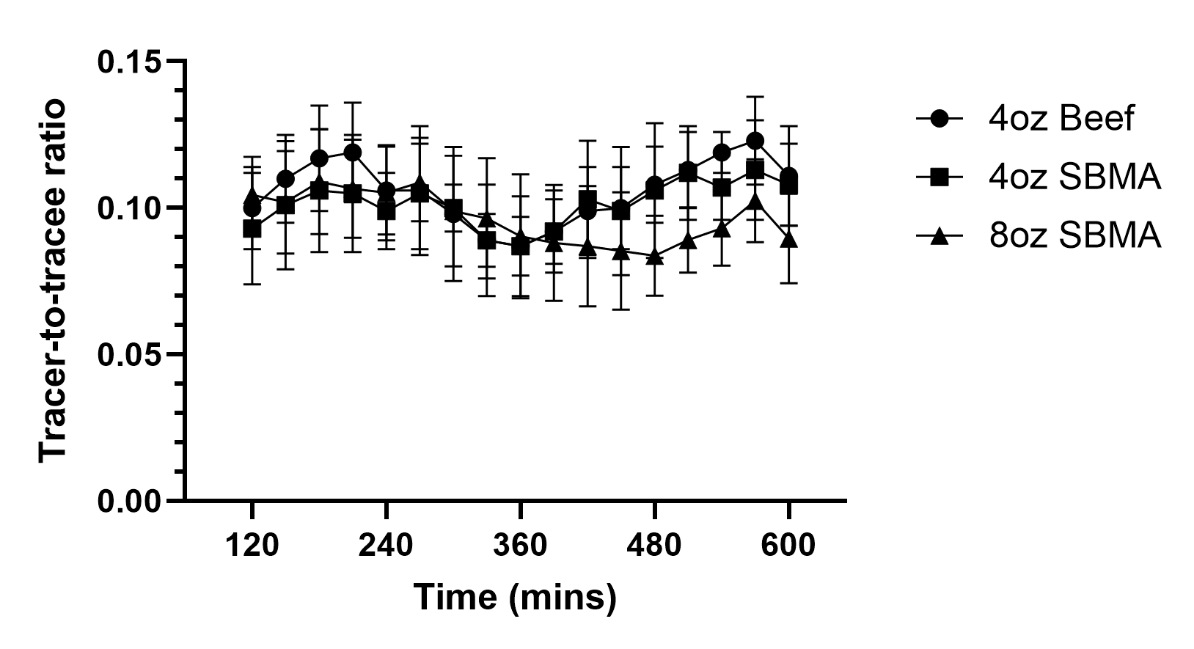


**Figure S1.** Plasma L-[ring-^2^H_5_] phenylalanine enrichment for each group (*n*=8 per group). Data presented as mean ± SD.

| **Table S1.** Ingredient composition of burger products | |
| --- | --- |
| Burger patty | Ingredients |
| Beef^a^ | Beef trimmings 80% lean, 20% fat. |
| Impossible^TM^ Burger^b^ | Water, soy protein concentrate, sunflower oil, coconut oil, natural flavors, 2% or less of: methylcellulose, cultured dextrose, food starch modified, yeast extract, soy leghemoglobin, salt, mixed tocopherols (antioxidant), l-tryptophan, soy protein isolate, vitamins and minerals [zinc gluconate, niacin, thiamine hydrochloride(vitamin b1), pyridoxine hydrochloride (vitamin b6), riboflavin (vitamin b2), vitamin b12]. |
| ^a^No other ingredients or binders were included in the beef burger patties according to federal regulations (9 CFR § 319).  ^b^Contains allium derivatives. Flavor made from heme, via fermentation of genetically engineered yeast. Mixed tocopherols as antioxidant. Binders: methylcellulose, food starch modified. | |
